# Waning effectiveness of mRNA COVID-19 vaccines against inpatient and emergency department encounters

**DOI:** 10.1371/journal.pone.0300198

**Published:** 2024-03-07

**Authors:** Theodoros V. Giannouchos, Nicole L. Hair, Bankole Olatosi, Xiaoming Li

**Affiliations:** 1 Department of Health Policy and Organization, The University of Alabama at Birmingham School of Public Health, Birmingham, AL, United States of America; 2 Department of Health Services Policy and Management, Arnold School of Public Health, University of South Carolina, Columbia, SC, United States of America; 3 Big Data Health Science Research Center, University of South Carolina, Columbia, South Carolina, United States of America; 4 Department of Health Promotion, Education, and Behavior, Arnold School of Public Health, University of South Carolina, Columbia, SC, United States of America; National Center for Global Health and Medicine, JAPAN

## Abstract

In the United States, most real-world estimates of COVID-19 vaccine effectiveness are based on data drawn from large health systems or sentinel populations. More data is needed to understand how the benefits of vaccination may vary across US populations with disparate risk profiles and policy contexts. We aimed to provide estimates of mRNA COVID-19 vaccine effectiveness against moderate and severe outcomes of COVID-19 based on state population-level data sources. Using statewide integrated administrative and clinical data and a test-negative case-control study design, we assessed mRNA COVID-19 vaccine effectiveness against SARS-CoV-2-related hospitalizations and emergency department visits among adults in South Carolina. We presented estimates of vaccine effectiveness at discrete time intervals for adults who received one, two or three doses of mRNA COVID-19 vaccine compared to adults who were unvaccinated. We also evaluated changes in vaccine effectiveness over time (waning) for the overall sample and in subgroups defined by age. We showed that while two doses of mRNA COVID-19 vaccine were initially highly effective, vaccine effectiveness waned as time elapsed since the second dose. Compared to protection against hospitalizations, protection against emergency department visits was found to wane more sharply. In all cases, a third dose of mRNA COVID-19 vaccine conferred significant gains in protection relative to waning protection after two doses. Further, over more than 120 days of follow-up, the data revealed relatively limited waning of vaccine effectiveness after a third dose of mRNA COVID-19 vaccine.

## Introduction

Messenger RNA (mRNA) vaccines are effective in preventing COVID-19 infection and virus-related complications [1,2]. Extended trials and real-world effectiveness studies have shown, however, that protection conferred by two doses of mRNA COVID-19 vaccine wanes over time [3–7]. There is accumulating evidence that a third (booster) dose substantially improves protection against infection as well as moderate and severe outcomes [8–10]. However, less is known about the durability of protection following a third dose. While some studies have reported that protection against severe COVID-19 remains high after several months [11,12], other work suggests that three-dose protection wanes rapidly [13]. Further, the benefits of mRNA COVID-19 vaccines and the extent to which those benefits wane may vary across subgroups with different underlying risk [6,14,15].

Analyses of national databases in Israel [4,16], Qatar [5], Sweden [6], and the United Kingdom [10,15] provide real-world population-level estimates of vaccine effectiveness (VE) and waning immunity. In the United States (US), most real-world evaluations of VE have been based data drawn from large regional or public health systems [13,14,17] or sentinel populations [9,18–20]. Population-level cohort studies in Indiana [21], New York [22], and North Carolina [23] are notable exceptions. However, these studies did not evaluate protection following receipt of a booster dose of mRNA vaccine, and only one such study [23] was conducted in the US South. More data is needed to understand how VE may vary across US regions with disparate risk profiles and policy contexts.

This study leverages statewide integrated administrative and clinical data and a rigorous test-negative case-control study design to generate population-based estimates of mRNA COVID-19 VE against SARS-CoV-2-related hospitalizations and emergency department (ED) visits among adults in South Carolina (SC). Our data span periods of Omicron as well as Delta (and pre-Delta) variant predominance. We focus on the durability of two-dose and three-dose protection. To this end, we present VE estimates at discrete time intervals for adults who received one, two or three doses of mRNA COVID-19 vaccine compared to adults who were unvaccinated. We evaluate changes in VE over time (waning) for the overall sample and in subgroups defined by age.

## Methods

### Data sources

This study used integrated data from two statewide databases with mandated reporting overseen by the SC Department of Health and Environmental Control–COVID-19 testing data and the State’s immunization registry–and a statewide all-payer claims database (hospital inpatient, outpatient, and ED). The SC Office of Revenue and Fiscal Affairs carried out the necessary data linkages and provided the research team with a de-identified dataset. The University of South Carolina Institutional Review Board determined that this study met the Not Human Subject criteria set forth by the Code of Federal Regulations (45 CFR 46). Since this study involved secondary analysis of existing data sources and no more than minimal risk, consent was waived for the study. The data were accessed on February 14, 2023.

### Study design

We used a retrospective test-negative case-control design to estimate mRNA VE against SARS-CoV-2-related hospitalizations and ED visits. The test-negative design is a widely applied statistical approach for monitoring VE in observational studies [24]. In order to mitigate potential biases resulting from differential care-seeking behaviors, we restricted the sample to patients who presented with COVID-like illness (CLI) [9,25–27]. VE was estimated by comparing the odds of prior COVID-19 vaccination among symptomatic patients who tested positive for SARS-CoV-2 (cases) with the odds among symptomatic patients who had a negative test result (controls). We estimated VE by number of vaccine doses and time elapsed since the last vaccine dose. Separate analyses were conducted for inpatients and ED patients.

### Study population and setting

The study population included SC adults aged ≥18 years with CLI who had a hospitalization or ED visit between 2 January 2021 and 23 April 2022 and had been tested for SARS-CoV-2 within the past 14 days. We excluded individuals who received ≥1 dose of non-mRNA COVID-19 vaccine. During our sample period, the BNT162b2 (Pfizer-BioNTech) and mRNA-1273 (Moderna) vaccines comprised the vast majority (96%) of doses administered in SC. Similar to previous work, we also excluded individuals who tested positive for SARS-CoV-2 >14 days or who received a first dose of mRNA vaccine <14 days or a third dose <7 days before the index encounter [9].

### Exposure

The exposure of interest was the receipt of one or more doses of mRNA COVID-19 vaccine. Patients were considered partially vaccinated if they had received one dose of mRNA vaccine ≥14 days or a second dose <14 days before the index encounter and vaccinated if they had received a second dose of mRNA vaccine ≥14 days before the index encounter. Fully vaccinated patients who received an additional (booster) dose ≥7 days before the index encounter were considered to have three-dose protection. Patients who had not yet received any doses of mRNA vaccine as of the index encounter date were considered unvaccinated.

To examine waning effectiveness, we further categorized vaccinated patients by time since vaccination (i.e., number of days between the last vaccine dose and the index encounter). For this purpose, we defined 60-day intervals ranging from ≤60 days since vaccination to >240 days or >120 days for the second and third dose, respectively. The shorter follow-up period for three-dose protection reflects the fact that, for most groups, a third dose of mRNA COVID-19 vaccine was not recommended until fall 2021.

### Outcome of interest and measurement

The primary outcome of interest was a positive or negative SARS-CoV-2 test result for an inpatient or ED patient with CLI. We considered results from tests performed within 14 days of the index encounter. Patients with laboratory-confirmed SARS-CoV-2 infection were classified as cases. Patients with negative SARS-CoV-2 test results were classified as controls. Cases could be included once in the inpatient or ED sample. Where cases had more than one encounter in the inpatient or ED setting, the index date was assigned using the encounter closest to the SARS-CoV-2 test. Controls could be included in the inpatient or ED samples multiple times [9].

### Covariates

Covariate selection was informed by known risk factors for COVID-19 infection and severe COVID-19 disease and data availability [28–30]. Demographic characteristics included age group, sex, race/ethnicity, insurance/payer type, and rural residence. Clinical characteristics included immunocompromised status and eight a priori identified underlying medical conditions: asthma, chronic obstructive pulmonary disease (COPD), cardiovascular disease, congestive heart failure, diabetes mellitus, renal failure, chronic liver disease, and neurological disease. Other variables included indicators corresponding to county, predominant variant, vaccine availability, and hospital teaching status.

### Statistical analysis

Separate analyses were conducted for inpatients and ED patients. For each setting, we described characteristics of SARS-CoV-2 positive cases and SARS-CoV-2 negative controls and compared them using the χ^2^ test for categorical variables and the two-sample t-test or Mann-Whitney U test for continuous variables. We analyzed the association between COVID-19 vaccination status and laboratory-confirmed SARS-CoV-2 infection using multivariable logistic regression models adjusted for potentially confounding demographic and clinical characteristics. Models were further conditioned on county, predominant variant (Omicron or Delta versus pre-Delta period), and vaccine availability (post-booster versus pre-booster period) such that case-control comparisons were made within the same region and period. We defined the pre-Delta, Delta, and Omicron periods using regional COVID-19 tracking data (reported weekly) and a 50% threshold for variant predominance.

Vaccination status was categorized by number of doses (one, two, or three doses of mRNA vaccine versus unvaccinated). Fully vaccinated patients were further categorized by time since last dose (measured in 60-day intervals). For each vaccination status indicator, we exponentiated the regression coefficient to obtain an adjusted odds ratio (AOR). We calculated VE = (1—AOR) x 100%. In secondary analyses, we stratified our sample according to age (18–44, 45–64 or ≥65) to assess VE and the extent of waning for subpopulations with different underlying risk. We also conducted separate analyses for the period September 2021 onward, when a third booster dose was recommended for most groups. Analyses were conducted using SAS software, version 9.4 (SAS Institute) and Stata software, version 17.0 (StataCorp).

## Results

### Characteristics of study population

Our sample included 37,344 hospitalizations and 79,454 ED visits (**Table 1**). The inpatient sample included 13,806 patients with a positive SARS-CoV-2 test result. Of those, 24.9%, 39.3%, and 35.7% were admitted during the Omicron, Delta, and pre-Delta periods, respectively. The ED sample included 26,271 patients who tested positive for SARS-CoV-2. Of those, 35.1%, 37.8%, and 27.0% were treated during the Omicron, Delta, and pre-Delta periods, respectively.

**Table 1:** Characteristics of adults who received care for COVID-like illness at a SC hospital or emergency department by SARS-CoV-2 test result.

	Hospital admissions				Emergency department visits			
	Total	SARS-CoV-2 negative	SARS-CoV-2 positive		Total	SARS-CoV-2 negative	SARS-CoV-2 positive	
Characteristics	(n = 37,344)	(n = 23,538)	(n = 13,806)		(n = 79,454)	(n = 53,183)	(n = 26,271)	
Variant time period^1^								
Pre-delta	42.1	45.8	35.7	<0.001	38.4	44.0	27.0	<0.001
Delta	35.6	33.4	39.3		36.7	36.2	37.8	
Omicron	22.3	20.7	24.9		24.9	19.9	35.1	
COVID-19 vaccination status								
Unvaccinated	65.5	56.9	80.3	<0.001	74.1	71.1	80.2	<0.001
Partially vaccinated^2^	5.4	6.3	3.9		4.6	5.1	3.6	
Two doses	23.6	29.9	13.1		18.0	19.9	14.2	
Time since second dose (%)				<0.001				<0.001
≤ 60 days	4.4	6.5	0.9		3.2	4.4	0.8	
61 to 120 days	4.8	6.8	1.4		3.6	4.4	2.2	
121 to 180 days	4.9	5.9	3.3		4.2	4.5	3.8	
181 to 240 days	4.4	5.2	2.9		3.4	3.6	2.9	
≥ 241 days	5.1	5.5	4.5		3.5	3.0	4.5	
Median days since encounter	153 (80–227)	138 (69–217)	194 (142–275)		149 (84–220)	136 (69–204)	188 (129–257)	
Three doses	5.4	6.9	2.7		3.3	3.9	2.0	
Time since third dose (%)				<0.001				<0.001
≤ 60 days	2.4	3.2	1.0		1.6	2.0	0.8	
61 to 120 days	2.1	2.6	1.2		1.3	1.5	0.9	
≥ 121 days	0.9	1.1	0.5		0.4	0.4	0.3	
Median days since encounter	66 (34–101)	65 (33–99)	79 (37–111)		62 (34–95)	59 (33–94)	70 (40–99)	
Mean (SD) length of stay (days)	7.1 (8.3)	6.3 (7.4)	8.5 (9.4)	<0.001	–	–	–	–
Age groups (years)								
18 to 34	4.8	4.7	5.1	<0.001	30.9	31.0	30.6	<0.001
35 to 49	10.8	8.9	14.1		24.1	22.4	27.7	
50 to 64	26.4	24.3	30.0		21.4	20.7	22.9	
65 to 70	36.9	37.7	35.4		16.7	18.0	14.2	
80 and older	21.1	24.4	15.5		6.8	8.0	4.6	
Sex								
Male	49.5	49.1	50.3	0.019	42.2	42.8	40.9	<0.001
Female	50.5	50.9	49.7		57.8	57.2	59.1	
Race/ethnicity								
Non-Hispanic White	68.2	69.7	65.6	<0.001	51.6	53.7	47.4	<0.001
Non-Hispanic Black	28.0	27.0	29.7		42.2	40.3	46.1	
Hispanic	1.7	1.4	2.3		2.7	2.7	2.8	
Other	2.1	1.9	2.4		3.4	3.3	3.6	
Insurance/payer type								
Private	16.0	11.9	22.9	<0.001	25.2	21.9	31.8	<0.001
Medicare	62.4	66.2	55.9		28.2	30.7	23.1	
Medicaid	7.3	7.6	6.9		16.4	16.5	16.2	
Uninsured	6.8	8.3	4.3		19.0	17.7	21.6	
Indigent/charitable organization	4.9	3.8	6.8		6.6	8.4	2.8	
Other	2.6	2.2	3.2		4.6	4.7	4.4	
Urban-rural residence								
Urban	68.2	67.7	69.1	0.004	67.8	67.5	68.5	0.006
Rural	31.8	32.3	30.9		32.2	32.5	31.5	
Underlying medical conditions								
Immunocompromised	14.5	16.1	11.8	<0.001	3.1	3.2	2.8	0.003
Asthma	5.2	4.7	6.1	<0.001	5.8	5.3	6.8	<0.001
COPD	28.6	30.7	24.9	<0.001	10.9	10.8	11.2	0.097
Cardiovascular disease	72.6	73.8	70.5	<0.001	33.4	33.2	33.7	0.11
Congestive heart failure	28.6	33.6	20.0	<0.001	4.3	4.7	3.3	<0.001
Diabetes mellitus	33.3	31.7	36.0	<0.001	13.2	12.8	14.1	<0.001
Renal failure	1.6	1.5	1.8	0.016	0.8	0.8	0.8	0.91
Chronic liver disease	5.0	5.4	4.2	<0.001	1.2	1.3	1.0	<0.001
Neurological disease	18.8	19.4	17.6	<0.001	1.7	1.9	1.4	<0.001
Teaching Hospital	43.7	43.8	43.4	0.51	27.5	28.7	25.1	<0.001

1. The pre-Delta (January 2, 2021 to July 2, 2021), Delta (July 3, 2021 to December 15, 2021), and Omicron (December 16, 2021 to April 23, 2022) periods were defined using regional COVID-19 tracking data (reported weekly) and a 50% threshold for variant predominance.

2. Patients were considered partially vaccinated if they had received one dose of mRNA vaccine at least 14 days before the index encounter or a second dose less than 14 days before the index encounter.

In the inpatient sample, 65.5% of patients were unvaccinated, while 23.6% were fully vaccinated with two doses of mRNA vaccine, and 5.4% had three-dose protection. The median time between the encounter date and most recent vaccination was 153 days (interquartile range (IQR): 80–227 days) and 66 days (IQR: 34–101 days) for the second and third dose, respectively. Most patients were non-Hispanic White (68.2%), had Medicare coverage (62.4%), and were ≥65 (57.9%). The most common underlying conditions among inpatients with CLI were cardiovascular disease (72.6%), diabetes mellitus (33.3%), congestive heart failure (28.6%), and COPD (28.6%); 14.5% had an immunocompromising condition. Compared to SARS-CoV-2 negative controls, patients who tested positive for SARS-CoV-2 were less than half as likely to have two-dose (13.1% versus 29.9%) or three-dose (2.7% versus 6.9%) protection, and significantly more likely to be unvaccinated (80.3% versus 56.9%). They were also less likely to have Medicare coverage (55.9% versus 66.2%), more likely to be privately insured (22.9% versus 11.9%) and had longer hospital stays (mean length of stay 8.5 versus 6.3 days).

In the ED sample, 74.1% of patients were unvaccinated, while 18.0% were fully vaccinated with two doses, and 3.3% had received a third dose of mRNA vaccine. The median time between the encounter date and the most recent vaccination 149 days (IQR: 84–220 days) and 62 days (IQR: 34–95 days) for the second and third dose, respectively. Compared to inpatients with CLI, ED patients were more racially and ethnically diverse, less likely to have Medicare coverage, and more likely be privately insured, uninsured, or have Medicaid coverage. They were also comparatively younger and had fewer underlying medical conditions. Most patients (55.0%) were under 50 years of age. Common underlying conditions included cardiovascular disease (33.4%), diabetes mellitus (13.2%), and COPD (10.9%); 3.1% of ED patients had an immunocompromising condition. SARS-CoV-2 positive cases were more likely than SARS-CoV-2 negative controls to be unvaccinated (80.2% versus 71.1%). They were also comparatively more likely to be non-Hispanic Black (46.1% versus 40.3%) or have private insurance coverage (31.8% versus 21.9%) and less likely to be non-Hispanic White (47.4% versus 53.7%) or have Medicare coverage (23.1% versus 30.7%).

### Vaccine effectiveness

**Fig 1:**
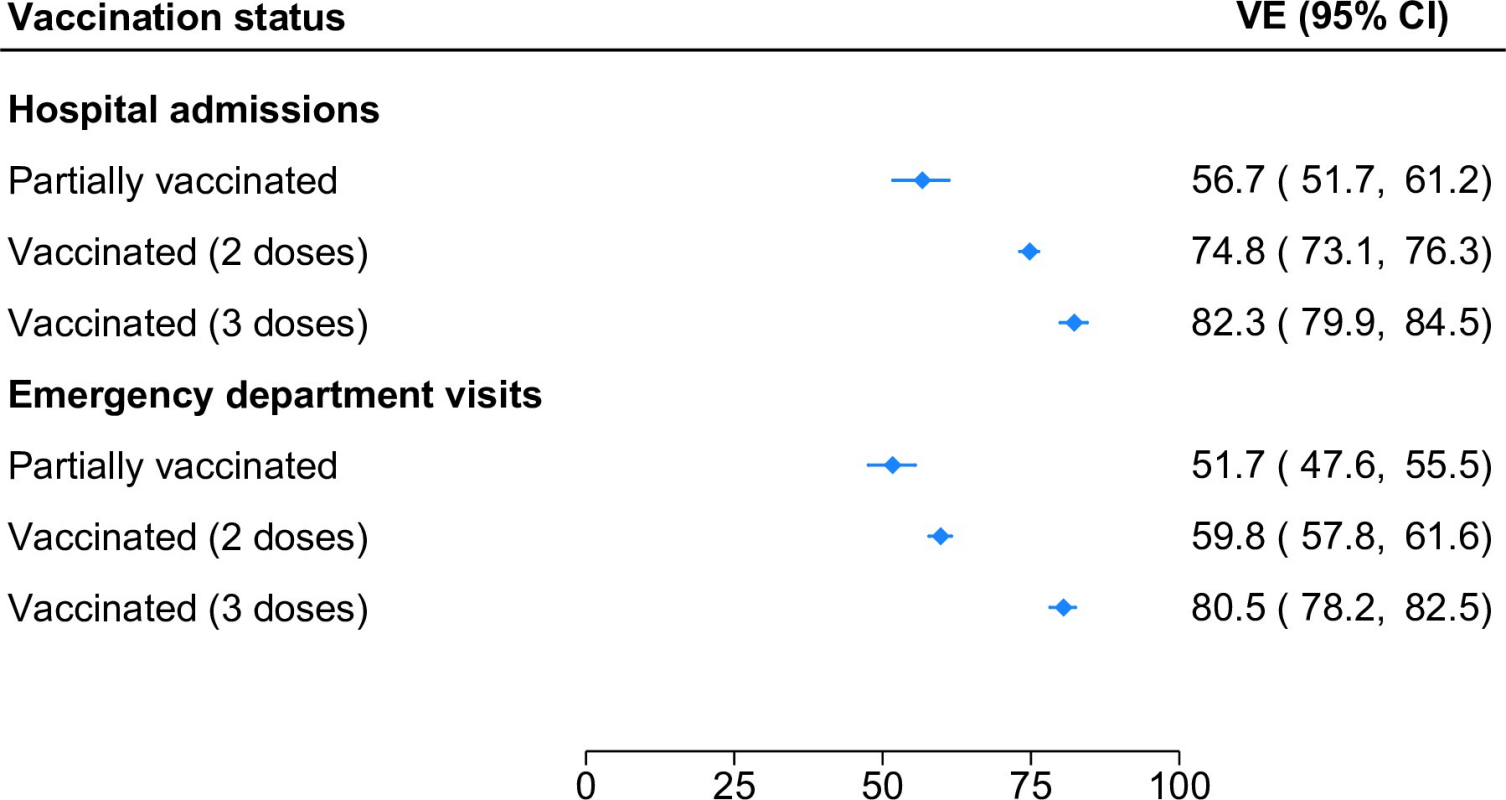
Effectiveness of mRNA COVID-19 vaccines against SARS-CoV-2 infection leading to hospital admission or emergency department visit by number of vaccine doses.

Estimates of VE from the inpatient and ED samples are shown in **Fig 1**. The effectiveness of partial vaccination against hospitalization was 56.7% (95% CI, 51.7%-61.2%), while the effectiveness of two-dose and three-dose vaccination was 74.8% (95% CI, 73.1%-76.3%) and 82.3% (95% CI, 79.9%-84.5%), respectively. Similar patterns were observed in the ED sample, although VE estimates were generally lower. With respect to ED visits, the effectiveness of partial vaccination, two-dose vaccination, and three-dose vaccination was 51.7% (95% CI, 47.6%-55.5%), 59.8% (95% CI, 57.8%-61.6%), and 80.5% (95% CI, 78.2%-82.5%), respectively.

**Fig 2:**
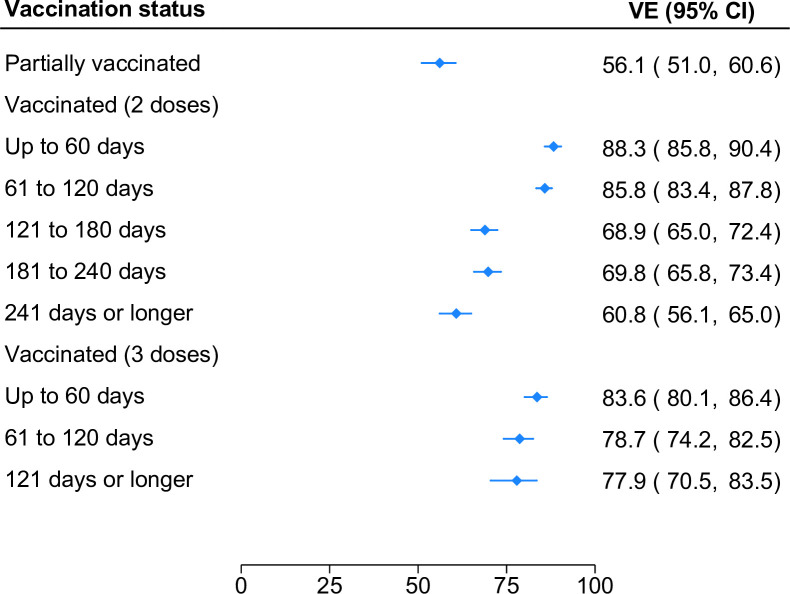
Effectiveness of mRNA COVID-19 vaccines against SARS-CoV-2 infection leading to hospital admission by number of vaccine doses and time since last dose.

VE against hospitalization waned over time (**Fig 2**). VE was highest ≤60 days following the second dose (88.3%; 95% CI, 85.8%-90.4%). Protection was similar at 61–120 days but then decreased moderately. VE was 68.9% (95% CI, 65.0%-72.4%), 69.8% (95% CI, 65.8%-73.4%), and 60.8% (95% CI, 56.1%-65.0%) at 121–180, 181–240, and ≥240 days from the second dose, respectively. Relatively high VE was restored following a third dose (83.6%; 95% CI, 80.1%-86.4%), with little evidence of waning ≥120 days from the third dose.

VE against ED visits was highest ≤60 days following the second dose (83.4%; 95% CI, 80.8%-85.6%) and then diminished steadily. VE was 68.7% (95% CI, 65.5%-71.5%), 52.0% (95% CI, 48.7%-55.8%), and 38.3% (95% CI, 32.6%-43.4%) at 61–120, 121–180, and ≥240 days from the second dose, respectively. However, like patterns reported in the inpatient sample, high VE was restored following a third dose (82.1%; 95% CI, 79.0%-84.7%). While there was little evidence of waning at ≥120 days from the third dose, it was not possible to assess potential waning at later time points.

In both the inpatient and ED sample, VE waned as time elapsed from the second dose; however, two-dose protection against ED visits waned more sharply. Comparing VE at ≤60 days and ≥240 days from the second dose, protection against ED visits decreased by 38.3 percentage points (pp; 95% CI 29.3–51.0 pp). Over the same time period, protection against hospital admissions decreased by just 27.5 pp (95% CI, 22.6–32.5 pp). The difference in the rate of waning (17.6 pp; 95% CI 10.1–25.1) is statistically significant.

### Vaccine effectiveness in subgroups

**Fig 3:**
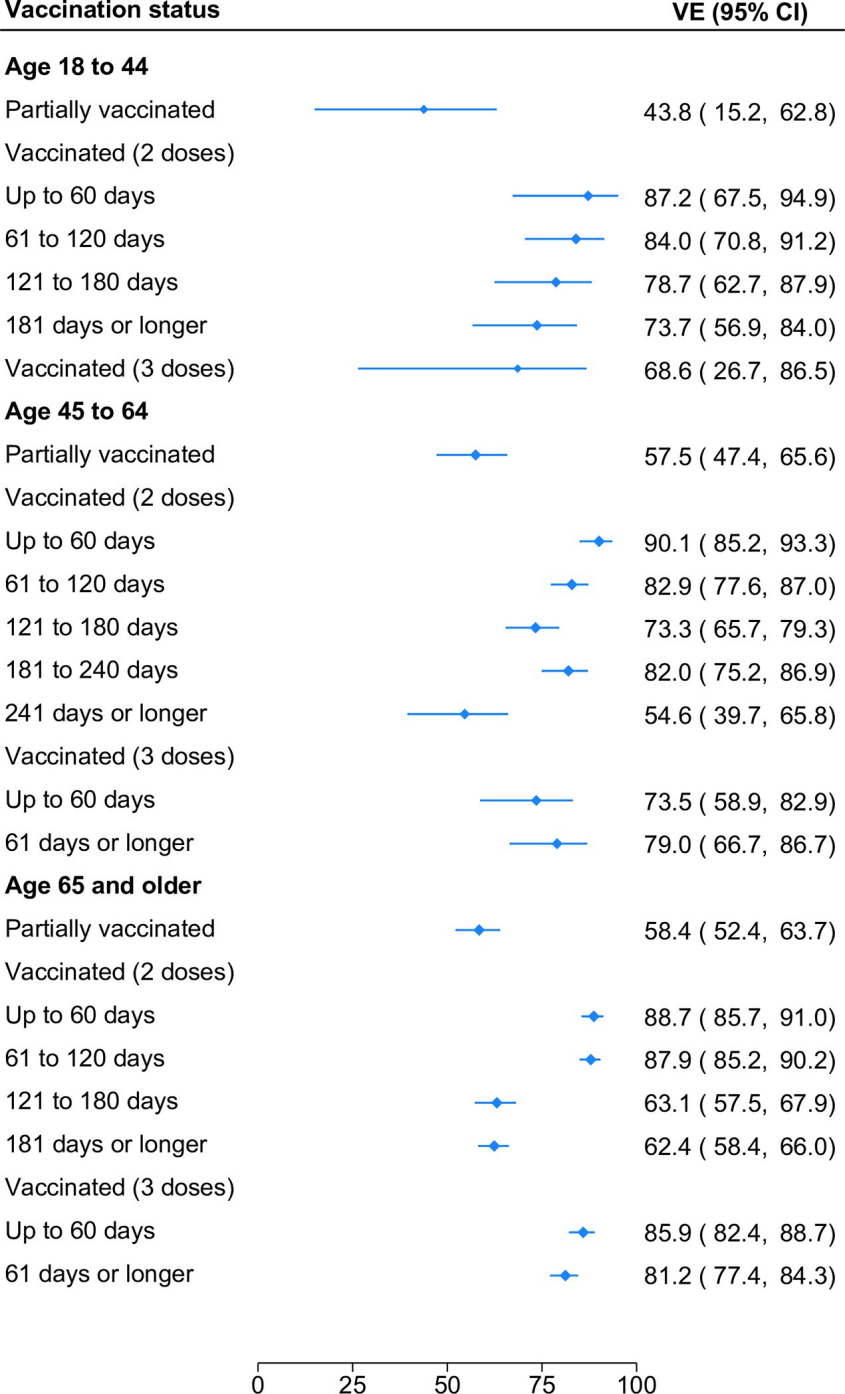
Effectiveness of mRNA COVID-19 vaccines against SARS-CoV-2 infection leading to hospital admission by age group, number of vaccine doses and time since last dose.

In secondary analyses, we stratified our sample according to patient age (18–44, 45–64, or ≥65). Within each age group considered, VE against hospitalizations waned as time elapsed since the second dose (**Fig 3**). Comparing VE ≤60 days following the second dose to VE at 121–180 days, two-dose protection waned from 87.2% (95% CI, 67.5%-94.9%) to 78.7% (95% CI, 62.7%-87.9%) among adults 18–44, from 90.1% (95% CI, 85.2%-93.3%) to 73.3% (95% CI, 65.7%-79.3%) among adults 45–64, and from 88.7% (95% CI, 85.7%-91.0%) to 63.1% (95% CI, 57.5%-67.9%) among adults ≥65. Among adults ≥45, high levels of protection against hospitalization were restored following a third dose (73.5%; 95% CI, 58.9%-82.9% and 85.9%; 95% CI, 82.4%-88.7% for adults 45–64 and ≥65, respectively). Owing to fewer cases in adults 18–44, three-dose protection against hospitalization in this age group was imprecisely estimated.

**Fig 4:**
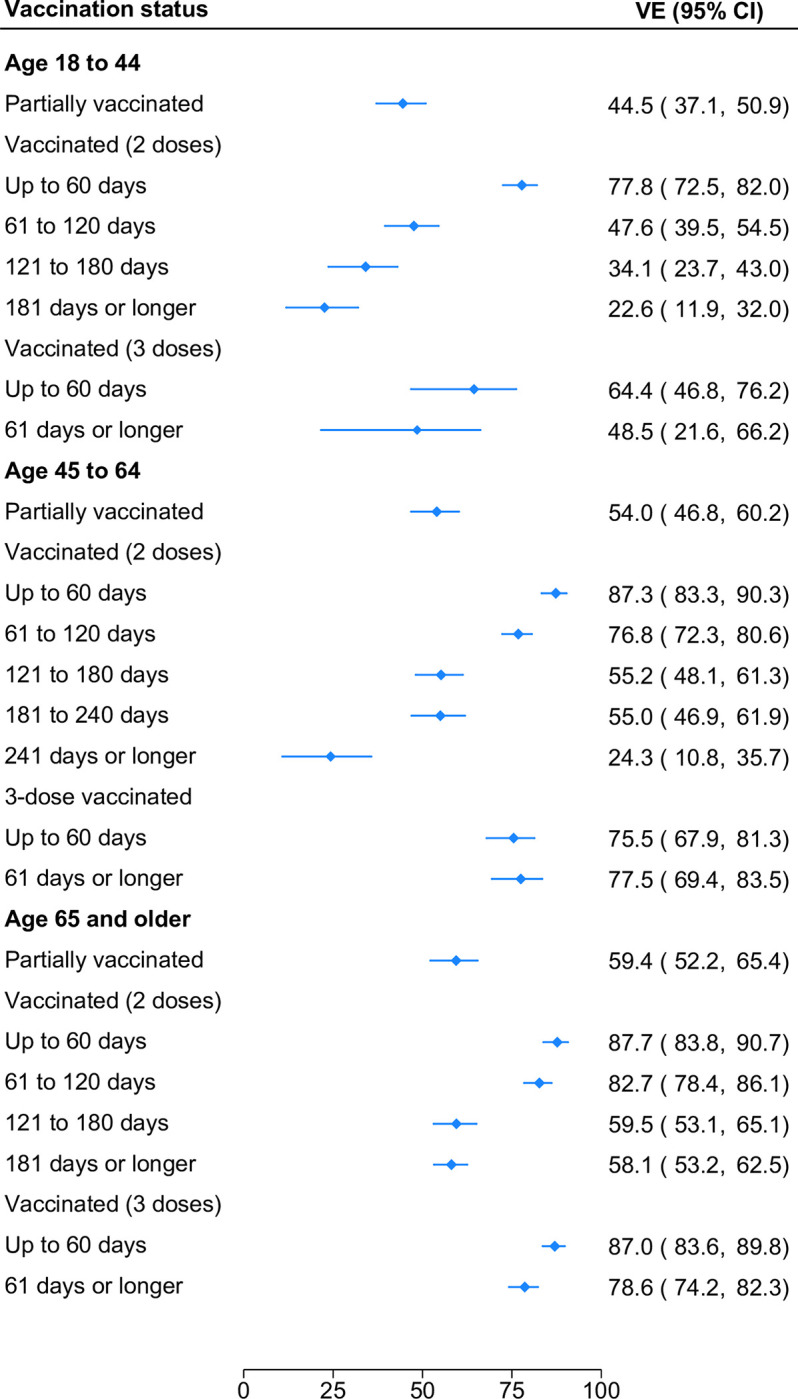
Effectiveness of mRNA COVID-19 vaccines against SARS-CoV-2 infection leading to emergency department visit by age group, number of vaccine doses and time since last dose.

In the ED sample (**Fig 4**), VE after two doses was lower and waned more sharply among younger age groups. For example, VE in adults 18–44 decreased by 55.2 pp (95% CI 44.2–66.1 pp) from 77.8% (95% CI, 72.5%-82.0%) at ≤60 days following the second dose to 22.6% (95% CI, 11.9%-32.0%) at ≥181 days, whereas VE in adults ≥65 decreased by 29.6 pp (95% CI 23.9–35.3 pp) from 87.7% (95% CI, 83.8%-90.7%) to 58.1% (95% CI, 53.2%-62.5%) over the same interval. The difference in the rate of waning (25.6 pp; 95% CI 13.2–37.9 pp) is statistically significant. Among adults ≥45, high levels of protection were restored following a third dose (75.5%; 95% CI, 67.9%-81.3% and 87.0%; 95% CI, 83.6%-89.8% for adults 45–64 and ≥65, respectively). Compared with estimates for older adults, the effectiveness of three-dose vaccination for adults 18–44 was lower (64.4%; 95% CI, 46.8%-76.2%). Even in this age group, however, a third dose of mRNA COVID-19 vaccine conferred significant gains in protection relative to waning vaccine protection after two doses.

### Sensitivity analyses

**Fig 5:**
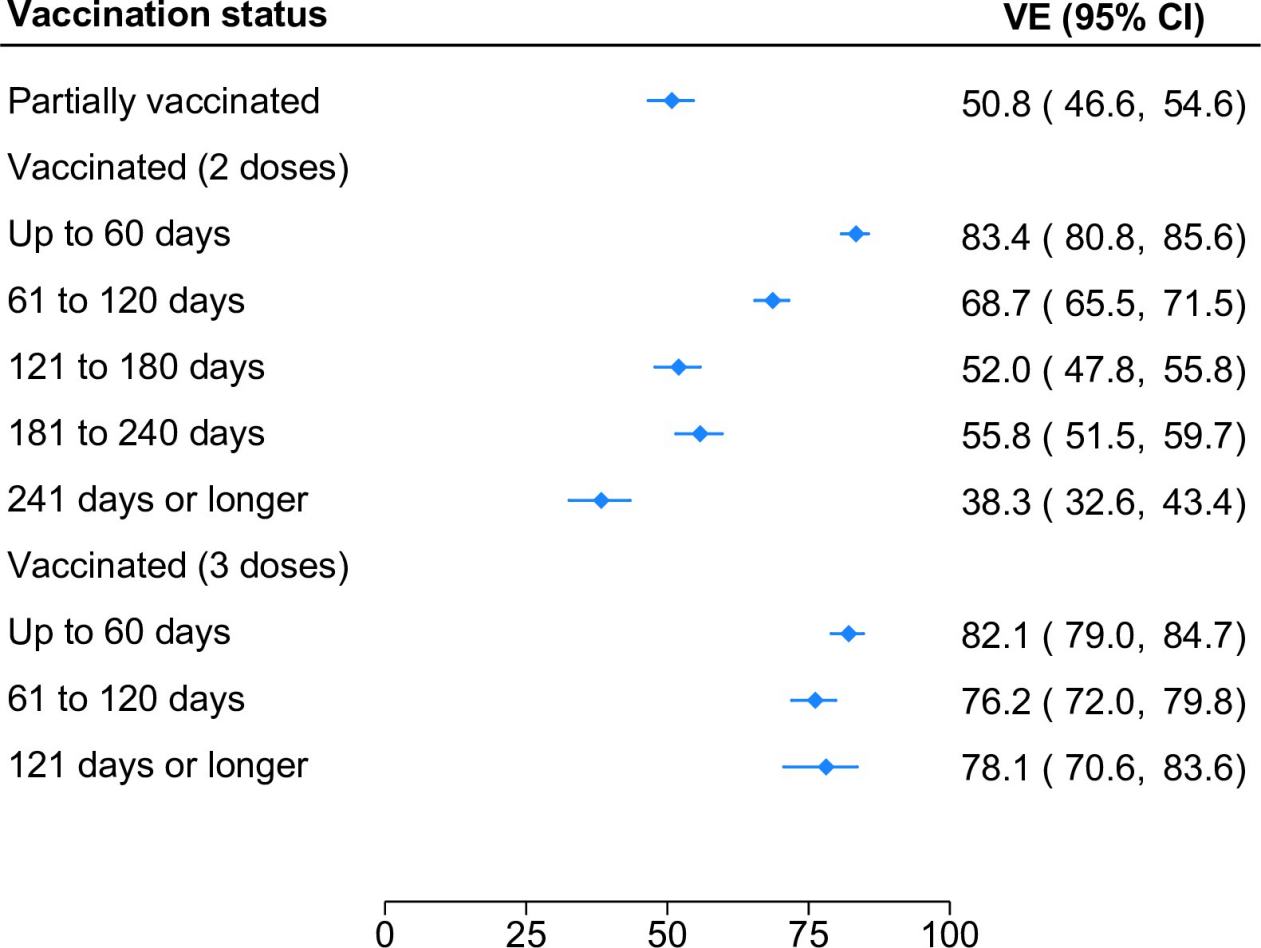
Effectiveness of mRNA COVID-19 vaccines against SARS-CoV-2 infection leading to emergency department visit by number of vaccine doses and time since last dose.

In supplemental analyses, we assessed VE from September 24, 2021, when a third booster dose was recommended for most groups, onward. During this period, the Delta and Omicron (from December 16, 2021) variants were predominant in SC. In both the inpatient (**S1 Fig**) and ED (**S2 Fig**) samples (see **S1 Table** for sample characteristics), patterns of waning VE following a second dose were similar to those reported for unrestricted models (Figs 2 and 5), though VE was generally lower. VE against hospitalizations waned from 76.7% (95% CI, 65.4%-84.2%) at ≤60 days to 56.5% (95% CI, 51.2%-61.2%) at >240 days. Over that same interval, two-dose protection against ED visits waned from 73.2% (95% CI, 67.1%-78.2%) to 20.2% (95% CI, 12.4%-27.4%). Following a third dose, VE against hospitalizations and ED visits increased substantially to 81.1% (95% CI, 77.1%-84.4%) and 76.0% (95% CI, 71.7%-79.6%), respectively. Further, we conducted separate analyses for periods when the Delta variant was predominant (July 3, 2021 to December 15, 2021) and when the Omicron variant was predominant (December 16, 2021 to April 23, 2022). In both the inpatient (**S3 Fig**) and ED (**S4 Fig**) samples, VE was greatly reduced with the emergence of the Omicron variant. However, in both the Delta period and the Omicron period, VE decreased over time. At ≤60 days from a second dose, VE against hospitalizations was 92.0% (95% CI, 87.7%, 94.8%) in the Delta period and 58.8% (95% CI, 26.8%, 76.8%) in the Omicron period. Two-dose protection against hospitalizations decreased to 74.0% (95% CI, 70.1%, 77.3%) and 49.1% (95% CI, 42.4%, 55.0%) at >181 days in the Delta and Omicron periods, respectively. Over that same interval, two-dose protection against ED visits waned from 88.4% (95% CI, 84.9%, 91.0%) and 73.7% (95% CI, 70.1%, 76.9%) in the Delta period and from 57.0% (95% CI, 44.3%, 66.8%) and 9.9% (95% CI, 1.5%, 17.6%) in the Omicron period. In all cases, a third dose restored relatively high levels of protection.

## Discussion

Two doses of mRNA COVID-19 vaccine were initially highly effective against SARS-CoV-2-related hospital admissions (88.3%; 95% CI, 85.8%-90.4%) and ED visits (83.4%; 95% CI, 80.8%-85.6%). Across all age groups, however, VE waned as time elapsed since the second dose. Compared to protection against hospitalization, protection against ED visits waned more sharply. In all cases, a third dose conferred significant gains in protection relative to waning protection after two doses. In the inpatient sample, VE was 83.6% (95% CI, 80.1%-86.4%) ≤60 days following a third dose. In the ED sample, VE after a third dose was 82.1% (95% CI, 79.0%-84.7%). Over more than 120 days of follow-up, waning of VE after a third dose was relatively limited.

Our estimates are broadly consistent with findings from other studies of real-world VE in the US and other countries. Two doses of mRNA COVID-19 vaccine were highly effective against moderate (e.g., ED visits) and severe (e.g., hospital admissions) outcomes of COVID-19. The effectiveness of two doses waned with time since vaccination [4–9,15]; however, a third dose substantially increased protection [8,9]. Consistent with prior work, we found two-dose vaccination to be more durably protective against severe outcomes of COVID-19 [5–9,15]. With respect to three-dose vaccination, we noted relatively limited waning of VE over more than 120 days (4 months) of follow-up. While some reports suggest that third-dose protection wanes rapidly [13,31], others have found that protection against SARS-CoV-2-related hospital admissions remains relatively high (VE above 75%) for at least 4 months [9,11,12].

One strength of our study is the use of integrated state-wide clinical and administrative data to generate population-based estimates of mRNA COVID-19 VE. To date, most real-world evaluations of VE against moderate or severe COVID-19 in the US have been based on analyses of electronic medical records from large regional or public health systems [13,14,17] or sentinel populations [9,18–20]. Another strength of our study is the application of a test-negative case-control study design. The test-negative design is a widely applied statistical approach for monitoring VE and is thought to minimize selection bias due to differences in health care access and health care-seeking behavior between vaccinated and unvaccinated patients [24]. Our models were further conditioned on county, predominant variant, and vaccine availability such that case-control comparisons were made within the same region and period. Finally, we were able to compare protection against severe (versus moderate) outcomes of COVID-19 in the overall sample as well as for different subgroups defined by age.

### Limitations

This study has some limitations. While the test-negative design is thought to reduce unmeasured confounding due to health care-seeking behavior, it is observational in nature and, therefore, subject to potential bias. Owing to our sample period, we were unable to assess longer-term protection of the third dose (i.e., beyond 4 or 5 months) or provide estimates of VE for updated bivalent mRNA COVID-19 vaccines (which were not widely available in the U.S. until fall 2022).

## Conclusion

The effectiveness of mRNA COVID-19 vaccines waned as time elapsed from the second dose; however, a third dose conferred significant gains in protection. These findings underscore the importance of mRNA vaccine booster doses during the Delta and Omicron periods and highlight the need for modernized monitoring and public health data systems to facilitate ongoing and more timely population-level estimates of COVID-19 VE in the US.

## Supporting information

S1 Fig Effectiveness of mRNA COVID-19 vaccines against SARS-CoV-2 infection leading to hospital admission during the post-booster period by number of vaccine doses and time since last dose. (TIF)

S2 Fig Effectiveness of mRNA COVID-19 vaccines against SARS-CoV-2 infection leading to emergency department visit during the post-booster period by number of vaccine doses and time since last dose. (TIF)

S3 Fig Effectiveness of mRNA COVID-19 vaccines against SARS-CoV-2 infection leading to hospital admission during the Delta and Omicron periods. (TIF)

S4 Fig Effectiveness of mRNA COVID-19 vaccines against SARS-CoV-2 infection leading to emergency department visit during the Delta and Omicron periods. (TIF)

S1 Table Characteristics of adults who received care for COVID-like illness at a SC hospital or emergency department by SARS-CoV-2 test result, September 24, 2021 to April 23, 2022. (DOCX)
