# Supplementary figures and images for: Waning effectiveness of mRNA COVID-19 vaccines against inpatient and emergency department encounters
Source: PLoS One. 2024 Mar 7;19(3):e0300198. doi: 10.1371/journal.pone.0300198 (PMC10919609; doi:10.1371/journal.pone.0300198)

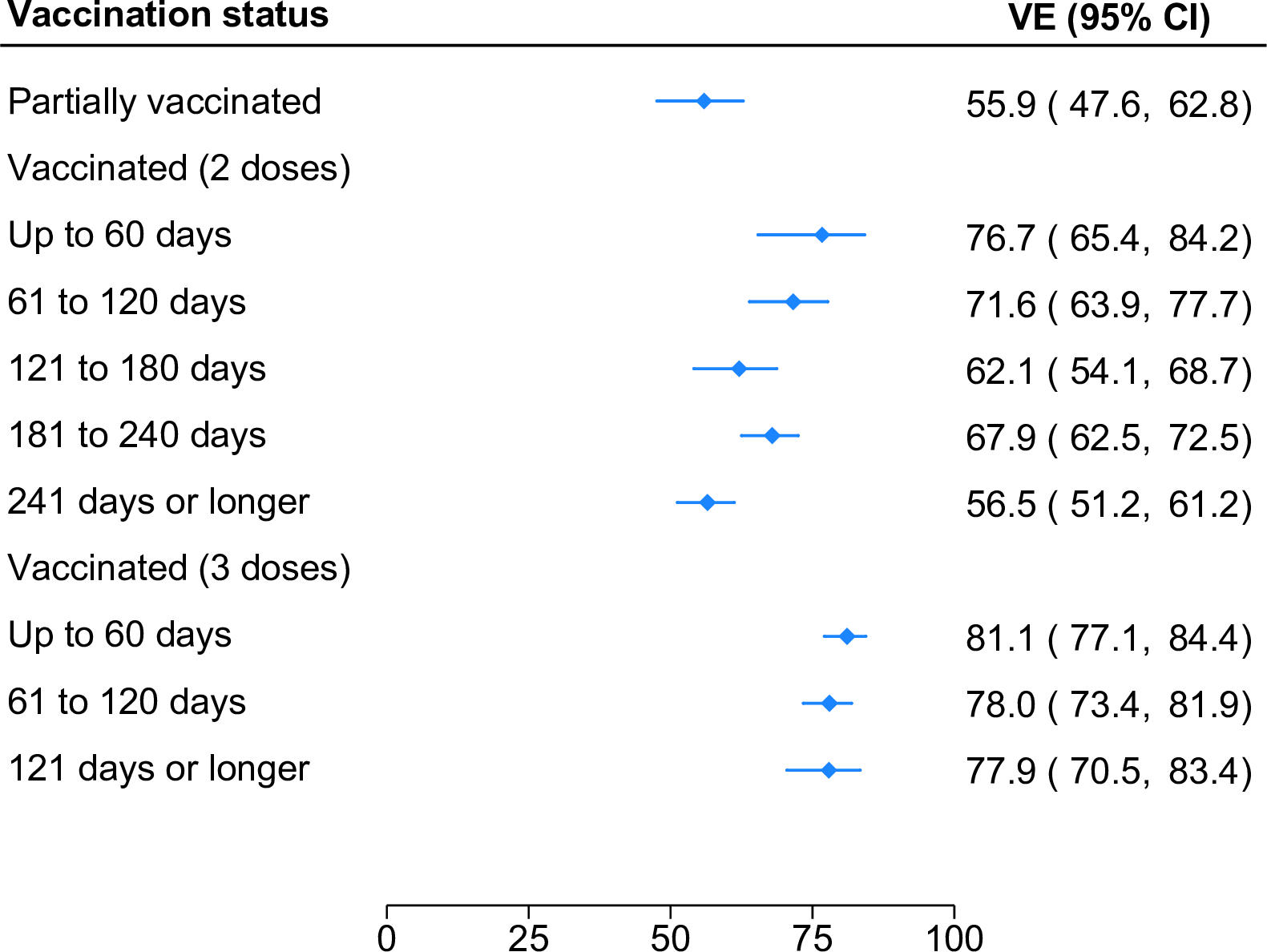

Supplement: S1 Fig — (TIF) [file pone.0300198.s001.tif]

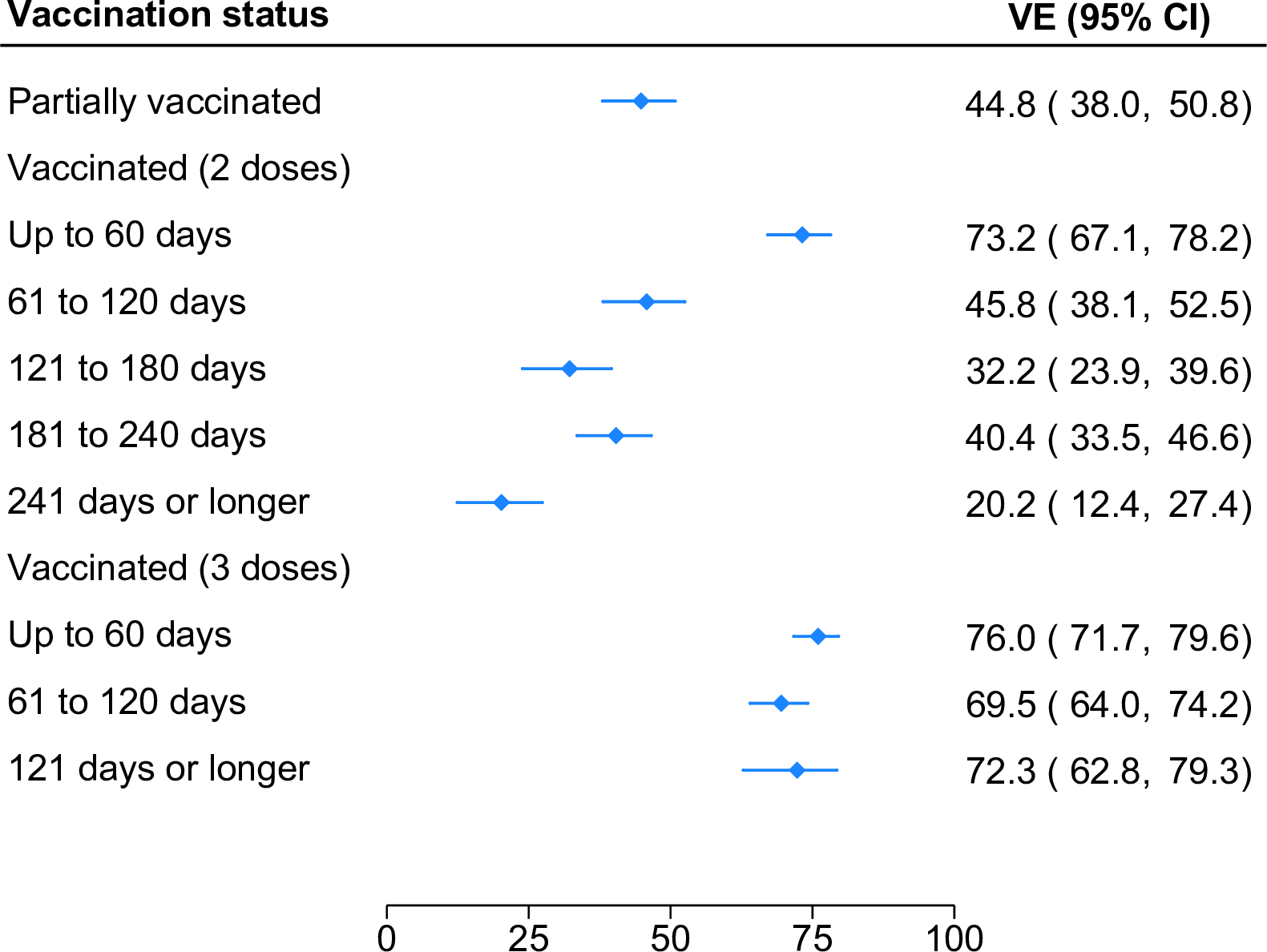

Supplement: S2 Fig — (TIF) [file pone.0300198.s002.tif]

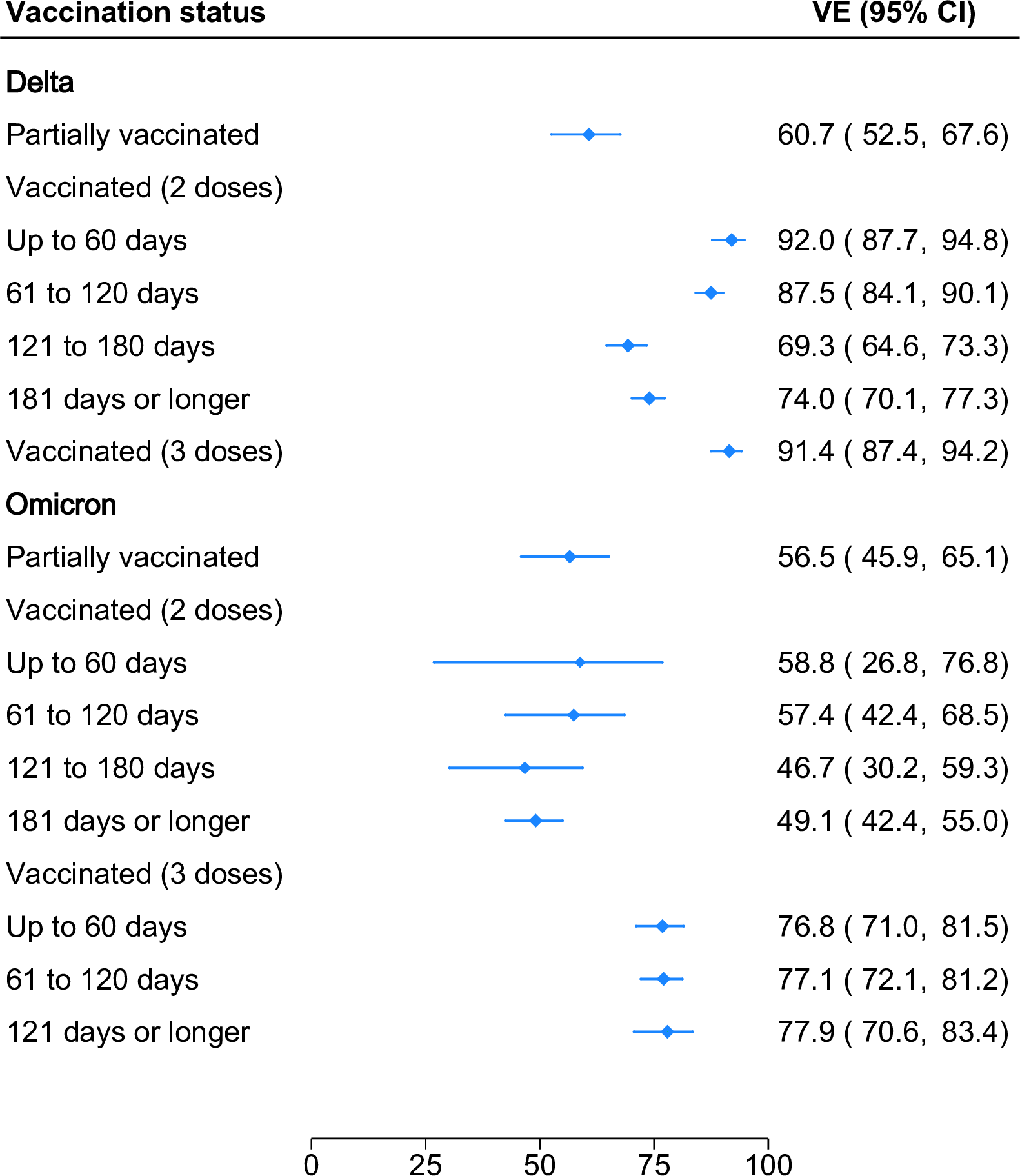

Supplement: S3 Fig — (TIF) [file pone.0300198.s003.tif]

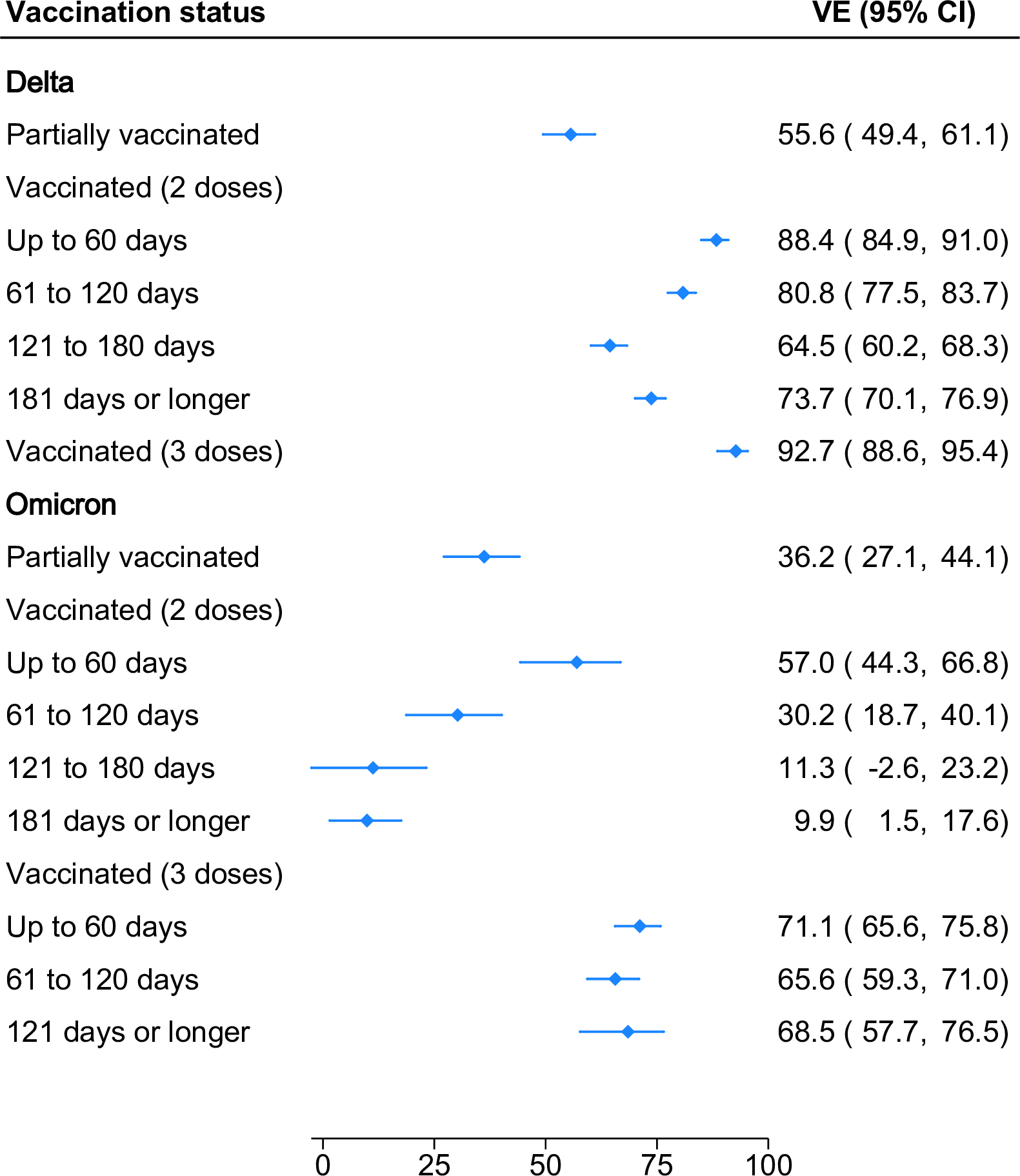

Supplement: S4 Fig — (TIF) [file pone.0300198.s004.tif]
