# Supplementary material for: Waning effectiveness of mRNA COVID-19 vaccines against inpatient and emergency department encounters
Source: PLoS One. 2024 Mar 7;19(3):e0300198. doi: 10.1371/journal.pone.0300198 (PMC10919609; doi:10.1371/journal.pone.0300198)
Supplement: S1 Table — (DOCX) [file pone.0300198.s005.docx]

Table S1. Characteristics of adults who received care for COVID-like illness at a SC hospital or emergency department by SARS-CoV-2 test result, September 24, 2021 to April 23, 2022

|  | **Hospital admissions** | | |  | **Emergency department visits** | | |  |
| --- | --- | --- | --- | --- | --- | --- | --- | --- |
|  | **Total** | **SARS-CoV-2 negative** | **SARS-CoV-2 positive** |  | **Total** | **SARS-CoV-2 negative** | **SARS-CoV-2 positive** |  |
| **Characteristics** | (n=14,109) | (n=8,927) | (n=5,182) |  | (n=31,062) | (n=19,499) | (n=11,563) |  |
| Variant time period^1^ |  |  |  |  |  |  |  |  |
| Pre-delta | --- | --- | --- | <0.001 | --- | --- | --- | <0.001 |
| Delta | 41.0 | 45.3 | 33.6 |  | 36.3 | 45.8 | 20.2 |  |
| Omicron | 59.0 | 54.7 | 66.4 |  | 63.7 | 54.2 | 79.8 |  |
| COVID-19 vaccination status |  |  |  |  |  |  |  |  |
| Unvaccinated | 49.5 | 39.9 | 65.9 | <0.001 | 58.7 | 54.3 | 66.2 | <0.001 |
| Partially vaccinated^2^ | 5.2 | 5.7 | 4.2 |  | 5.5 | 6.0 | 4.6 |  |
| Two doses | 31.3 | 36.3 | 22.7 |  | 27.5 | 29.0 | 24.8 |  |
| Time since second dose (%) |  |  |  | <0.001 |  |  |  | <0.001 |
| ≤ 60 days | 1.5 | 1.9 | 0.6 |  | 2.3 | 3.1 | 1.0 |  |
| 61 to 120 days | 2.9 | 3.6 | 1.8 |  | 3.7 | 3.9 | 3.3 |  |
| 121 to 180 days | 4.7 | 5.5 | 3.3 |  | 5.5 | 5.7 | 5.1 |  |
| 181 to 240 days | 8.6 | 10.8 | 4.9 |  | 7.0 | 8.1 | 5.2 |  |
| ≥ 241 days | 13.6 | 14.5 | 12.0 |  | 9.0 | 8.3 | 10.3 |  |
| Median days since encounter | 227 (168-280) | 223 (164-271) | 248 (178-300) |  | 201 (130-258) | 194 (124-248) | 221 (140-271) |  |
| Three doses | 14.1 | 18.1 | 7.2 |  | 8.3 | 10.6 | 4.4 |  |
| Time since third dose (%) |  |  |  | <0.001 |  |  |  | <0.001 |
| ≤ 60 days | 6.3 | 8.3 | 2.8 |  | 4.0 | 5.4 | 1.8 |  |
| 61 to 120 days | 5.5 | 6.9 | 3.2 |  | 3.3 | 4.1 | 2.1 |  |
| ≥ 121 days | 2.3 | 2.9 | 1.3 |  | 1.0 | 1.2 | 0.5 |  |
| Median days since encounter | 67 (35-102) | 65 (35-99) | 79 (37-111) |  | 62 (35-95) | 60 (33-94) | 70 (40-100) |  |
| Mean (SD) length of stay (days) | 7.1 (8.2) | 6.5 (7.4) | 8.2 (9.2) | <0.001 | – | – | – | – |
| Age groups (years) |  |  |  |  |  |  |  |  |
| 18 to 34 | 4.5 | 4.5 | 4.5 | <0.001 | 31.4 | 30.2 | 33.5 | <0.001 |
| 35 to 49 | 9.3 | 8.3 | 10.9 |  | 23.9 | 21.5 | 27.9 |  |
| 50 to 64 | 25.5 | 23.6 | 28.8 |  | 20.4 | 20.2 | 20.8 |  |
| 65 to 70 | 38.0 | 38.2 | 37.7 |  | 17.1 | 19.2 | 13.5 |  |
| 80 and older | 22.7 | 25.4 | 18.0 |  | 7.2 | 8.9 | 4.3 |  |
| Sex |  |  |  |  |  |  |  |  |
| Male | 50.0 | 49.4 | 51.0 | 0.070 | 42.1 | 43.6 | 39.6 | <0.001 |
| Female | 50.0 | 50.6 | 49.0 |  | 57.9 | 56.4 | 60.4 |  |
| Race/ethnicity |  |  |  |  |  |  |  |  |
| Non-Hispanic White | 70.7 | 71.8 | 69.0 | 0.003 | 51.8 | 54.7 | 46.8 | <0.001 |
| Non-Hispanic Black | 25.5 | 24.7 | 26.8 |  | 41.8 | 38.5 | 47.3 |  |
| Hispanic | 1.8 | 1.6 | 2.1 |  | 2.9 | 3.2 | 2.6 |  |
| Other | 2.0 | 1.9 | 2.1 |  | 3.5 | 3.6 | 3.3 |  |
| Insurance/payer type |  |  |  |  |  |  |  |  |
| Private | 14.2 | 11.7 | 18.4 | <0.001 | 24.5 | 20.9 | 30.4 | <0.001 |
| Medicare | 64.6 | 66.5 | 61.5 |  | 28.5 | 32.0 | 22.6 |  |
| Medicaid | 7.1 | 7.2 | 6.9 |  | 16.8 | 15.9 | 18.3 |  |
| Uninsured | 7.1 | 8.5 | 4.5 |  | 20.2 | 19.1 | 22.0 |  |
| Indigent/charitable organization | 4.7 | 3.9 | 6.2 |  | 5.8 | 7.7 | 2.5 |  |
| Other | 2.3 | 2.3 | 2.4 |  | 4.3 | 4.4 | 4.1 |  |
| Urban-rural residence |  |  |  |  |  |  |  |  |
| Urban | 68.9 | 69.1 | 68.6 | 0.510 | 68.2 | 68.6 | 67.5 | 0.045 |
| Rural | 31.1 | 30.9 | 31.4 |  | 31.8 | 31.4 | 32.5 |  |
| Underlying medical conditions |  |  |  |  |  |  |  |  |
| Immunocompromised | 15.1 | 15.8 | 13.8 | 0.001 | 3.1 | 3.2 | 3.0 | 0.425 |
| Asthma | 5.0 | 4.8 | 5.3 | 0.223 | 6.0 | 5.4 | 6.9 | <0.001 |
| COPD | 29.1 | 30.0 | 27.5 | 0.002 | 11.3 | 11.2 | 11.4 | 0.525 |
| Cardiovascular disease | 73.1 | 73.7 | 72.1 | 0.032 | 33.7 | 34.2 | 32.8 | 0.013 |
| Congestive heart failure | 29.9 | 33.5 | 23.8 | <0.001 | 4.2 | 4.6 | 3.5 | <0.001 |
| Diabetes mellitus | 31.9 | 30.1 | 35.1 | <0.001 | 13.3 | 13.4 | 13.3 | 0.945 |
| Renal failure | 1.6 | 1.2 | 2.1 | 0.016 | 0.7 | 0.6 | 0.8 | 0.91 |
| Chronic liver disease | 5.0 | 5.1 | 4.7 | 0.331 | 1.2 | 1.3 | 1.1 | 0.157 |
| Neurological disease | 20.0 | 19.8 | 20.4 | 0.391 | 1.8 | 2.0 | 1.6 | 0.016 |
| Teaching Hospital | 43.4 | 43.3 | 43.5 | 0.812 | 27.5 | 28.4 | 26.0 | <0.001 |

1. The pre-Delta (January 2, 2021 to July 2, 2021), Delta (July 3, 2021 to December 15, 2021), and Omicron (December 16, 2021 to April 23, 2022) periods were defined using regional COVID-19 tracking data (reported weekly) and a 50% threshold for variant predominance.

2. Patients were considered partially vaccinated if they had received one dose of mRNA vaccine at least 14 days before the index encounter or a second dose less than 14 days before the index encounter.
